# Supplementary material for: Ultrasound-Triggered Release of 5-Fluorouracil from Soy Lecithin Echogenic Liposomes
Source: Pharmaceutics. 2021 Jun 1;13(6):821. doi: 10.3390/pharmaceutics13060821 (PMC8229429; doi:10.3390/pharmaceutics13060821)
Supplement: Supplementary file 1 [file pharmaceutics-13-00821-s001.zip › pharmaceutics-1119007-supplementary.pdf]

Supplementary Materials: Ultrasound-Triggered Release of 5-fluorouracil from Soy Lecithin Echogenic Liposomes

Charles Izuchukwu Ezekiel, Alain Murhimalika Bapolisi, Roderick Bryan Walker and Rui Werner Maçedo Krause

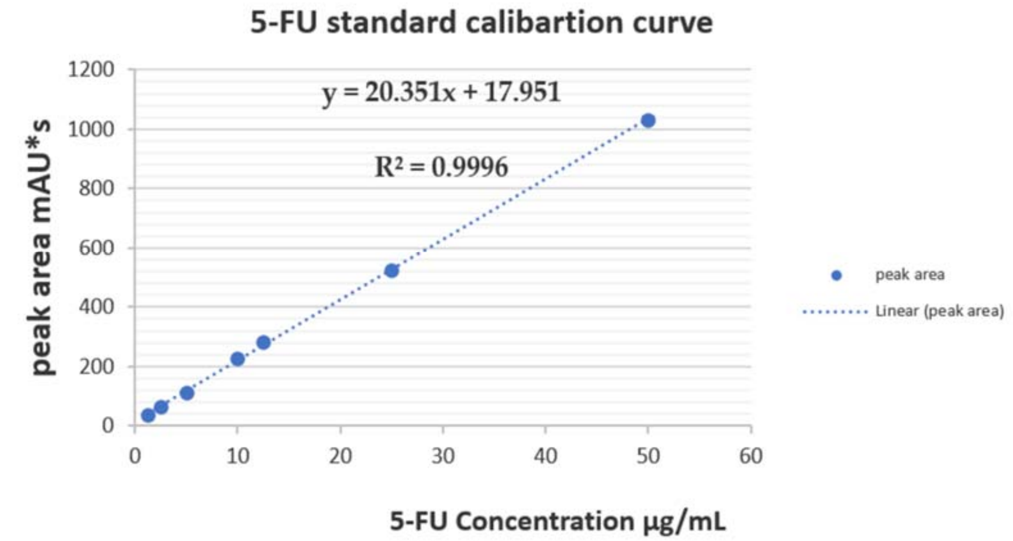

Figure 1. 5-Fluorouracil (5-FU) standard plot under the concentration range of 1.25-50 µg/mL.

Table 1. Standard deviation and recovery values for the 5 days analysis.

Concentration (µg/mL)	Intraday precision (%RSD)	Interday precision (%RSD)	Accuracy (% Recovery)
1.25	1.28	0.98	96.22
2.5	1.33	1.63	94.51
5	0.84	1.62	96.29
10	0.52	3.22	104.36
12.5	1.25	1.15	104.98
25	1.22	1.04	99.52
50	1.21	0.94	99.14
